# Genomic Analyses for Selective Signatures and Genes Involved in Hot Adaptation Among Indigenous Chickens From Different Tropical Climate Regions

**DOI:** 10.3389/fgene.2022.906447

**Published:** 2022-07-22

**Authors:** Nai-Yi Xu, Zhen-Yu Liu, Qi-Meng Yang, Pei-Pei Bian, Ming Li, Xin Zhao

**Affiliations:** ^1^ Key Laboratory of Animal Genetics, Breeding and Reproduction of Shaanxi Province, College of Animal Science and Technology, Northwest A&F University, Yangling, China; ^2^ Department of Biology, University of Konstanz, Konstanz, Germany; ^3^ Department of Animal Science, McGill University, Montreal, QC, Canada

**Keywords:** indigenous chicken, tropical climate, selection signature, hot adaptation, parallelism

## Abstract

Climate change, especially weather extremes like extreme cold or extreme hot, is a major challenge for global livestock. One of the animal breeding goals for sustainable livestock production should be to breed animals with excellent climate adaptability. Indigenous livestock and poultry are well adapted to the local climate, and they are good resources to study the genetic footprints and mechanism of the resilience to weather extremes. In order to identify selection signatures and genes that might be involved in hot adaptation in indigenous chickens from different tropical climates, we conducted a genomic analysis of 65 indigenous chickens that inhabit different climates. Several important unique positively selected genes (PSGs) were identified for each local chicken group by the cross-population extended haplotype homozygosity (XP-EHH). These PSGs, verified by composite likelihood ratio, genetic differentiation index, nucleotide diversity, Tajima’s D, and decorrelated composite of multiple signals, are related to nerve regulation, vascular function, immune function, lipid metabolism, kidney development, and function, which are involved in thermoregulation and hot adaptation. However, one common PSG was detected for all three tropical groups of chickens via XP-EHH but was not confirmed by other five types of selective sweep analyses. These results suggest that the hot adaptability of indigenous chickens from different tropical climate regions has evolved in parallel by taking different pathways with different sets of genes. The results from our study have provided reasonable explanations and insights for the rapid adaptation of chickens to diverse tropical climates and provide practical values for poultry breeding.

## Introduction

Climate change is one of the major threats facing the global livestock industry. In particular, extreme weather, including heat waves and cold waves, are not only challenging animal survival, health, production, and reproduction performance but also adversely affecting animal ecosystems, such as quality and quantity of feed and forage and safety of water source (Rojas-Downing et al., 2017; Rashamol and Sejian, 2018). At the same time, the demand for animal products, especially poultry products, is increasing. Furthermore, concerns for food safety, animal welfare, and environmental impact have put additional pressure on the animal husbandry industry. Thus, rearing animal breeds with high productivity and good environmental adaptability is desirable. Indigenous livestock and poultry with strong environmental adaptability because of long time evolution lay the foundation for achieving this goal. Considering the characteristics of wide distribution, largest population size, and short growth periods, chickens can also be used as ideal models to study genetic adaptations to environments (Lawler, 2016).

The domestic chickens were mainly derived from the red jungle fowl (RJF) subspecies *Gallus gallus spadiceus*, despite other junglefowl species also contributing genetically (Wang et al., 2020). Because of their portability and strong fecundity, domestic chickens have spread all over the world and are now the largest number of domestic animals raised globally (Lawler, 2016). Both humans and the environment have played fundamental roles in the evolutionary process of domestic chickens. Under natural and artificial selections, chickens have evolved genetic adaptations to different climate zones, including tropical, temperate, and even frigid climates. Although commercial chickens have become the mainstream of breeding, local chickens still play an important role in rural regions, especially in developing countries. Growth, development, and reproduction traits of indigenous village chickens have been relatively well studied (Dana et al., 2011; Haunshi et al., 2011; Moazeni et al., 2016). However, the excellent environmental adaptability of these indigenous village chickens remains to be explored.

Very few studies have been carried out to research the hot adaptability of native chickens (Lawal et al., 2018; Asadollahpour Nanaei et al., 2022). There is a need for comprehensive studies on the genetic mechanisms of domestic chickens adapting to naturally occurring hot humid and hot arid environments (e.g., tropical rainforest climate, tropical monsoon climate, and tropical desert climate). Indonesia belongs to the typical tropical rainforest climate, with high temperatures and rainfall throughout the year. Its annual average temperature is 25–27°C, and its annual rainfall is 1600–2200 mm (Lee, 2015). India has the typical tropical monsoon climate, with high temperatures and seasonal rainfall. Its annual average temperature is above 22°C, and annual rainfall mainly occurs in summer (June–September). Furthermore, the annual mean temperature increased by 0.22°C per decade from 1971 to 2003 (Kothawale and Rupa Kumar, 2005). The climate of the Arabian Peninsula is semi-arid and arid. The annual mean temperature of Saudi Arabia varies from 25°C to 38°C. In Saudi Arabia, extreme hot and dry climate has been persistent, with extremes increasing in magnitude and frequency over the past 15 years (Almazroui et al., 2014). Northern China mainly belongs to the temperate continental climate and temperate monsoon climate. The temperature varies distinctly in different seasons and precipitation is at a low level throughout the year with uneven seasonal distribution and being concentrated in summer (Domrös and Peng, 2012). Therefore, we performed a genomic analysis of 65 indigenous chickens from Indonesia, India, Saudi Arabia, and northern China, which represent three different tropical climate zones and one temperate climate zone.

## Materials and Methods

### Sample Collection and Whole-Genome Sequence Quality Control

In this study, we downloaded 65 whole-genome resequencing data of indigenous chickens from the NCBI database (Supplementary Table S1), including 20 Indonesian chickens, 20 Indian chickens, 5 Saudi Arabian chickens, and 20 chickens of northern China (7 Bian chickens, 7 Dagu chickens, and 6 BeijingYou chickens) (Yi et al., 2014; Lawal et al., 2018; Wang et al., 2020). Except for the above groups, we also collected worldwide whole-genome resequencing data of other chickens, including *Gallus gallus spadiceus* from China and Thailand, Chantecler chickens, Tibetan chickens, chickens of southern China, commercial chickens (Cornish, European broiler, White Plymouth Rock, Rhode Island Red, and White Leghorn), Iranian chickens, and Ethiopian chickens (Supplementary Table S1), for haplotype analysis and nonsynonymous mutation analysis (Fan et al., 2013; Yi et al., 2014; Wang M.-S. et al., 2015; Li et al., 2017; Lawal et al., 2018; Wang et al., 2020; Xu et al., 2021). To filter out low-quality reads and adapter sequences of raw data, Trimmomatic v0.36 was used based on default parameters (Bolger et al., 2014).

### Sequence Alignment and Variants Calling

The high-quality sequences were aligned to the chicken reference genome (GRCg6a) using the Burrows–Wheeler Aligner “BWA-MEM” algorithm with conventional parameters (Li and Durbin, 2009). Then, all reads were sorted and merged, and all duplicate reads were removed using Picard tools with default command and parameters. Next, the genome analysis toolkit (GATK, v3.6-0-g89b7209) was used to call single nucleotide polymorphisms (SNPs) (McKenna et al., 2010). The SNPs that met the following criteria were included: 1) <1/3 × mean sequencing depth (over all included individuals) < 3×; 2) mapping quality (MQ) > 40.0; 3) Quality by Depth (QD) > 2.0; 4) Fisher Strand (FS) > 60.0; and 5) MQRankSum>−12.5; 6) ReadPosRankSum>−8. The identified SNPs of all chicken samples were phased using BEAGLE v4 (Browning and Browning, 2007) with default parameters.

### Annotation of Genomic Variants

Annotation of all SNPs was implemented via ANNOVAR using the GRCg6a database (Wang et al., 2010). According to genome annotations, SNPs are located in the following regions: exonic regions, intronic regions, intergenic regions, splicing, 3′ untranslated region, 5′ untranslated region, upstream, and downstream. The functional categorization of SNPs includes synonymous, nonsynonymous, stopgain, and stoploss.

### Population Structure Analysis

Neighbor-joining (NJ) tree, principal component analysis (PCA), and ADMIXTURE analysis were used to explore the genetic relationships among chicken populations. Using the PLINK software (Purcell et al., 2007), we constructed an individual-based NJ tree based on the matrix of pairwise genetic distances from the autosomal SNPs of 65 chickens. The tree was visualized using iTOL (Letunic and Bork, 2019). In addition, PLINK was used for pruning pairs with --indep-pairwise 50 10 0.2 to reduce SNP redundancy caused by linkage disequilibrium. Based on pruned SNPs, PCA was performed using EIGENSOFT v5.052 with SmartPCA and eigenvectors’ significance was detected by the Tracy–Widom test (Patterson et al., 2006). Then, the figures were plotted using the first (PC1) and second (PC2) principal components with R packages. The population genetic structure was estimated based on genome-wide unlinked SNPs using the model-based assignment program ADMIXTURE v1.3.0 (Alexander et al., 2009). ADMIXTURE was run for each possible group number with 200 bootstrap replicates.

### Selective Sweep Analysis

Under the combined action of natural selection and artificial selection, animals will develop a variety of phenotypes and traits. During the formation of these traits, selection pressure will leave an imprint on the genome regions, showing high EHH, high composite likelihood ratio (CLR), high genetic differentiation index (*F*st), low nucleotide diversity (π), and negative Tajima’s D value. Here, we performed a genome-wide selective signal detection using the cross-population extended haplotype homozygosity (XP-EHH) analysis, which was used to detect nearly or ongoing fixed selective sweeps signature (Sabeti et al., 2007). XP-EHH values for every SNP were calculated using the default settings of selscan v1.1 (Szpiech and Hernandez, 2014), which was designed to detect ongoing or nearly fixed selective sweeps by comparing haplotypes of two populations. The XP-EHH value is directional. A positive value suggests selection in the candidate group, whereas a negative value suggests selection in the reference group. In our XP-EHH analysis, the average normalized XP-EHH score was counted in each 40-kb sliding window with a 20-kb step. The windows with the top 1% of XP-EHH value, which was obtained by the outlier method, were regarded as candidate positively selected regions, and the protein-coding genes annotated in the outlier were considered as candidate positively selected genes (PSGs). Breeds with a similar level of domestication and a distinct level of environmental temperature in comparison with candidate groups could be considered as the best reference group. The XP-EHH analysis was carried out with Indonesian, Indian, and Saudi Arabian chickens, which represent tropical rainforest climate, tropical monsoon climate, and tropical desert climate, as the candidate populations versus a reference group from temperate continental climate and temperate monsoon climate regions (chickens of northern China). In addition, we combined Indonesian, Indian and Saudi Arabian chickens into a group as the candidate group, and conducted an XP-EHH analysis with the reference group chickens of northern China. To further validate candidate positively selected regions and PSGs, four other methods were used to carry out region selective analyses with a 4 kb sliding window and a 2 kb increment: 1) CLR is a typical method, which is based on frequency spectrum to detect selective sweep signature (Nielsen et al., 2005). 2) *F*st is a common indicator of the degree of differentiation between groups (Weir and Cockerham, 1984). 3) *π* is often used to estimate the diversity of a population. 4) Tajima’s D is a statistical method for testing the neutral mutation, which could distinguish DNA sequences with random and nonrandom evolution (Tajima, 1989). We calculated *F*st, π, and Tajima’s D using vcftools (Danecek et al., 2011) with default parameters and detected CLR using sweepFinder2 (Degiorgio et al., 2016) with default parameters. We performed a statistic, decorrelated composite of multiple signals (DCMS), to combine several statistics (XP-EHH, CLR, and *F*st) while accounting for the respective correlation. The calculation of the DCMS value draws on the method of the previous study (Ma et al., 2015). Moreover, *p* values were estimated based on Z-transformed values using the standard normal distribution.

Haplotype analysis plays an important role in exploring causative genes by detecting haplotype diversity in the candidate regions or genes. Therefore, haplotype analysis and nonsynonymous mutation analysis were conducted to further confirm the PSGs in our study. We used in-house Perl scripts to extract genome-wide haplotype information based on the above phased file and further plotted haplotype figures using R packages. We searched for nonsynonymous mutation sites of PSGs on the GRCg6a gff file and then calculated the frequency of homozygous mutant, heterozygous mutant, and homozygous wild type of this site in each population based on the phased file.

## Results

### Characterization of Genomic Variants

**FIGURE 1:**
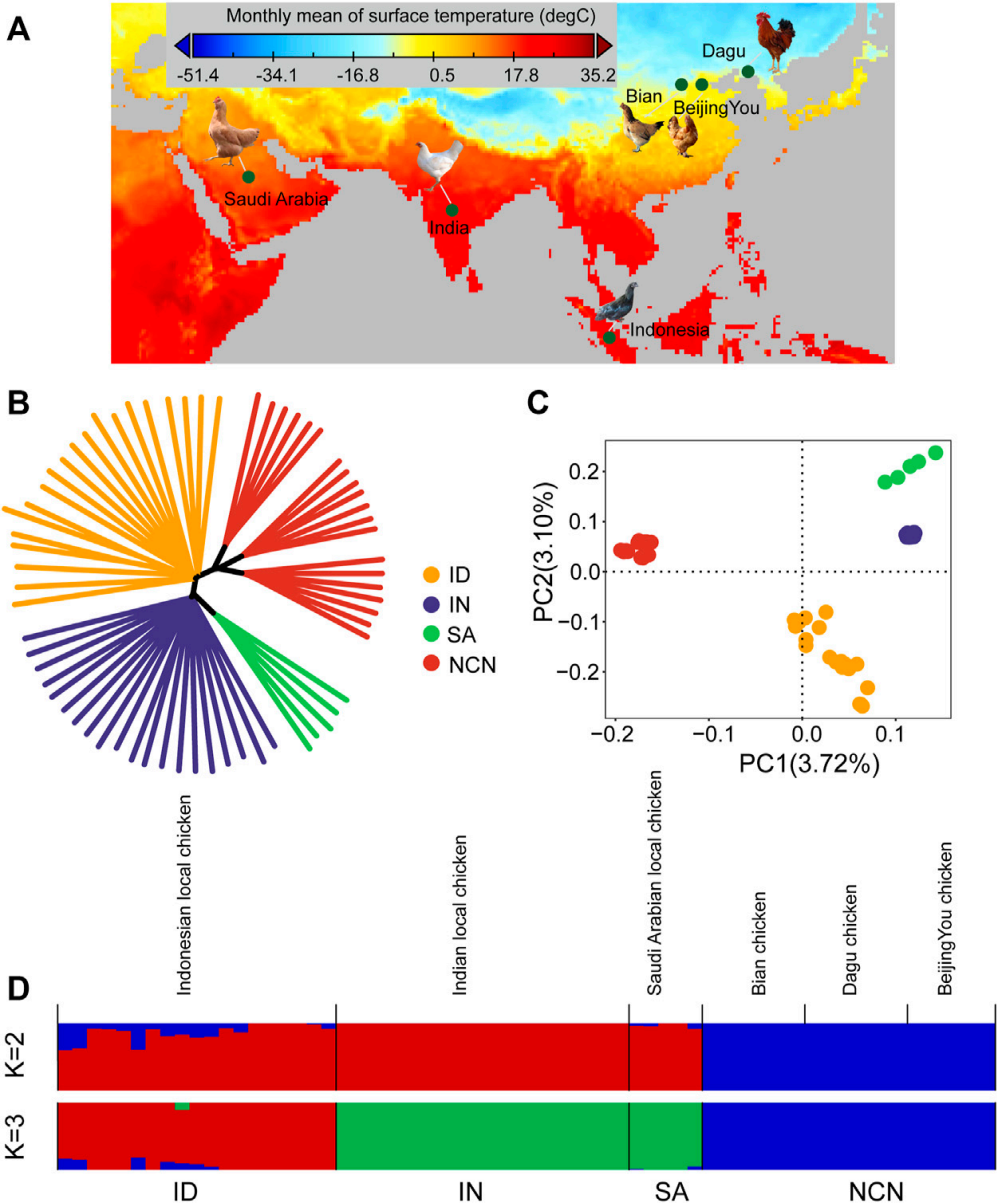
Population structure and relationships of tropical chickens. **(A)** geographic variation of monthly mean surface temperature for the location of chickens used in the study. **(B)** neighbor-joining tree. **(C)** principal component analysis (PCA) with the first (PC1) and second (PC2) principal components. **(D)** genetic structure of all chicken groups using ADMIXTURE program with K = 2 and K = 3. Indonesian chickens (ID), Indian chickens (IN), Saudi Arabian chickens (SA), and chickens of northern China (NCN).

Sixty-five chicken genomes from three different tropical climate zones and one temperate climatic zone were filtered for quality checks and adapter pollutions, and the high-quality genome sequences were mapped to GRCg6a, resulting in an average of 99.35% coverage and 6.40× depth (Figure 1A, Supplementary Table S1). For SNPs in each breed, the Indonesian chickens (10,851,370) and Indian chickens (10,298,801) displayed more SNPs, whereas the SNPs were less for chickens of northern China (8,992,252) and Saudi Arabian chickens (8,543,050) (Supplementary Table S2). Almost half of the SNPs of each breed were located in intronic regions, followed by intergenic regions. Moreover, the exon regions contained approximately 1.6% of the total SNPs in each breed with nonsynonymous, synonymous, stop–gain, and stop–loss. As expected, there are some SNPs within downstream, upstream, 3′ untranslated region, and 5′ untranslated region, which may regulate gene expression (Supplementary Table S2).

### Population Structure Analysis

To explore relatedness among chickens that inhabit different tropical climates, we performed NJ tree, PCA, and ADMIXTURE analysis using autosomal SNPs of the 65 chickens. The NJ tree showed that each breed clustered together (Figure 1B). The PCA showed that the PC1 was driven by differences between chickens of northern China and other chickens, and the PC2 separates Indonesian chickens and other two chicken groups (Indian chickens and Saudi Arabian chickens) (Figure 1C). In genetic clustering analysis, we used the ADMIXTURE program, where K is the assumed number of ancestral populations. The results of the admixture corroborated the finding in PCA. When *K* = 2, the chickens were genetically divided into chickens of northern China and the remaining groups. When *K* = 3, the Saudi Arabian chickens shared similar ancestries with Indian chickens (Figure 1D).

### Selection Sweeps Signals in Indonesian Chickens

**FIGURE 2:**
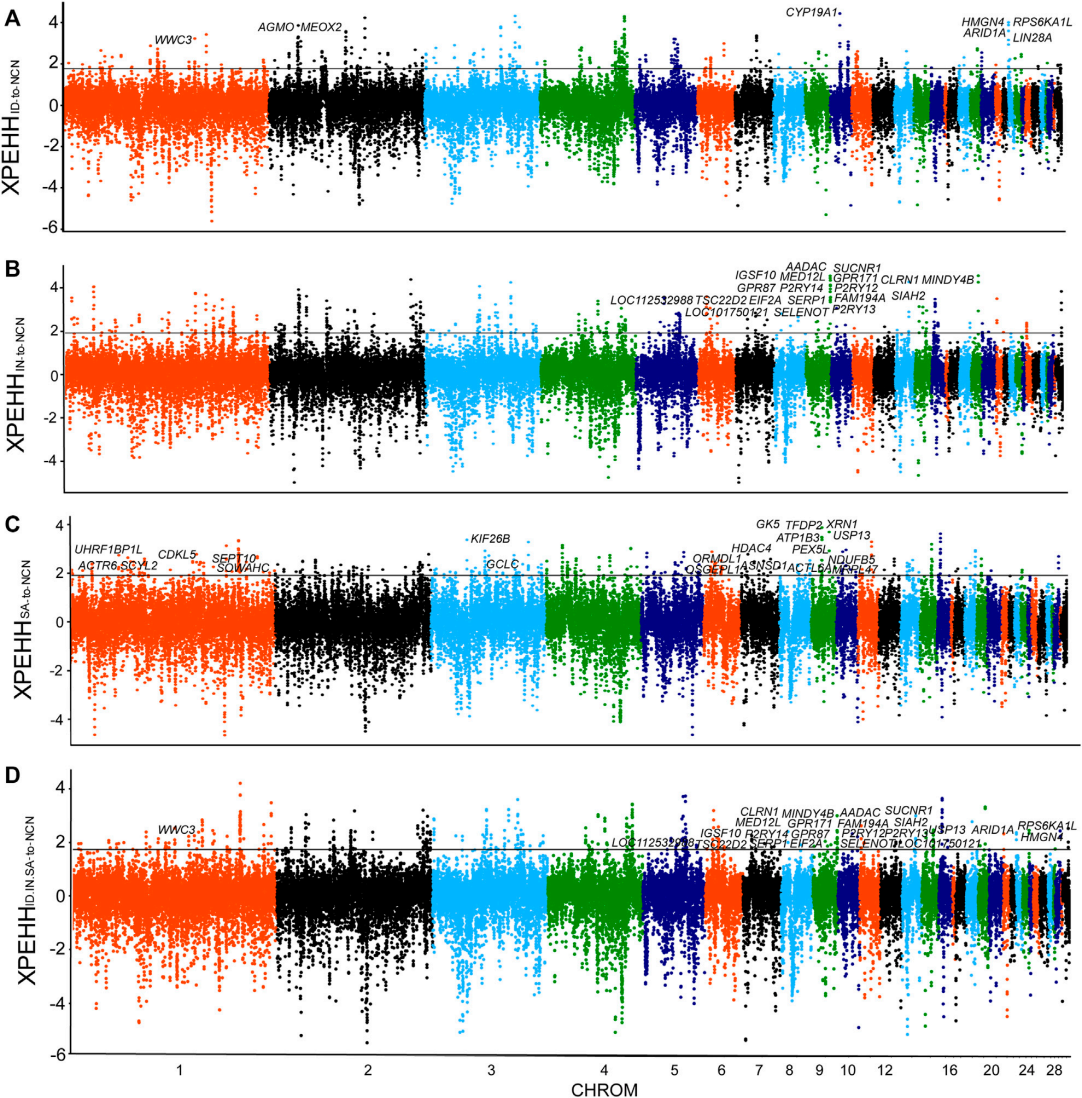
Selection Sweeps analysis. **(A)** XP-EHH analysis (ID chickens to NCN chickens). **(B)** XP-EHH analysis (IN chickens to NCN chickens). **(C)** XP-EHH analysis (SA chickens to NCN chickens). **(D)** XP-EHH analysis (ID.IN.SA chickens to NCN chickens). Sliding window analyses with 40-kb window and 20-kb increment, using 99th percentile cutoff.

**FIGURE 3:**
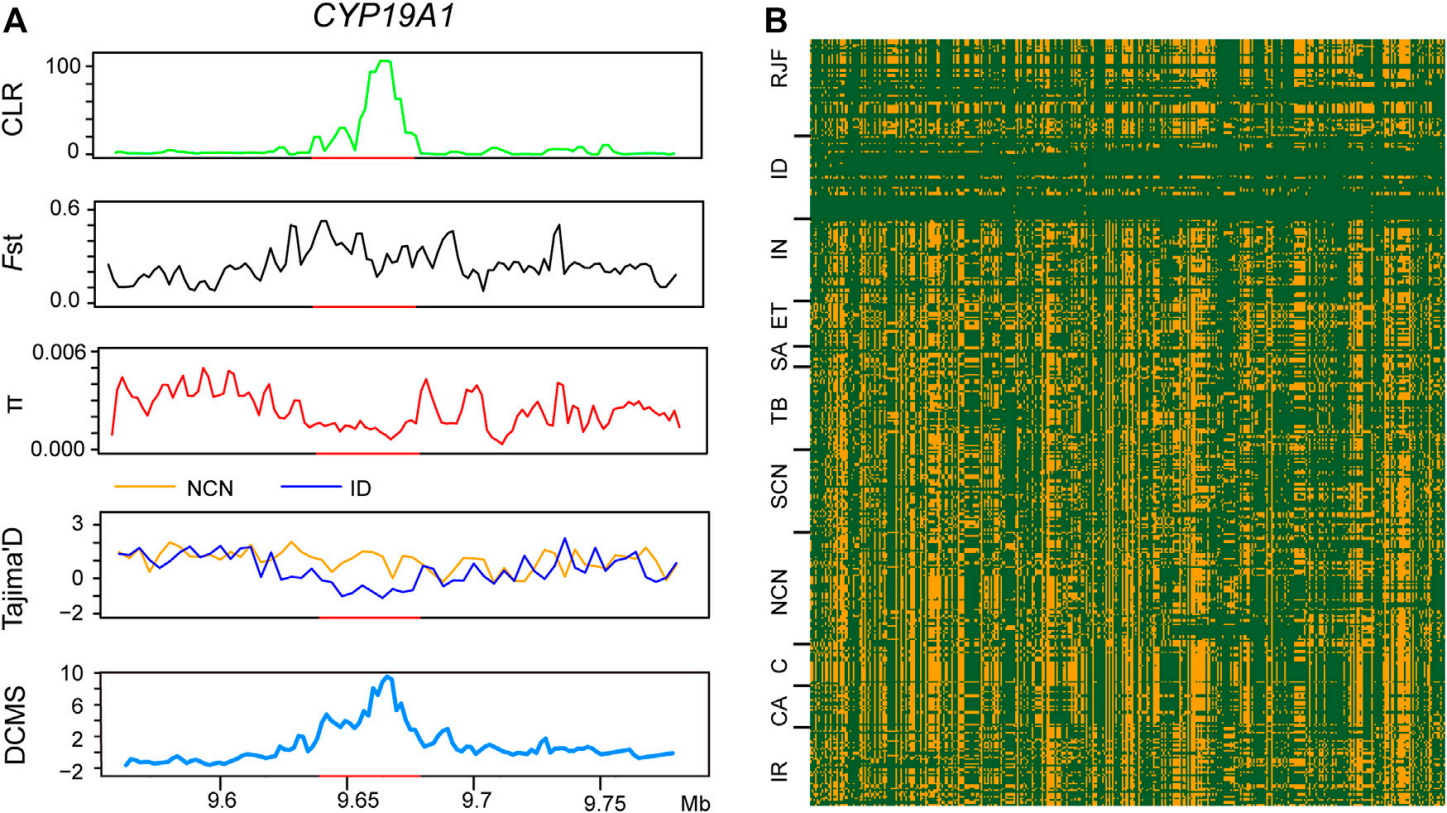
Selective signatures and haplotype analysis in *CYP19A1* gene identified by Indonesian chickens. **(A)** the *CYP19A1* gene is additionally validated by CLR, *F*st, π, Tajima’s D, and DCMS. **(B)** haplotype analysis at the *CYP19A1* gene. *Gallus gallus spadiceus* from China and Thailand (RJF), Chantecler chickens (CA), Tibetan chicken (TB), chickens of southern China (SCN), commercial chickens (C), Iranian chickens (IR), and Ethiopian chickens (ET).

The Indonesian, Indian, and Saudi Arabian chickens are well adapted to tropical climates. Although they all inhabit hot climates, the climate characteristics, such as solar radiation, diurnal temperature differences, and precipitation, of these regions vary greatly. To reveal genomic sweep footprints in these chickens, we performed XP-EHH analysis of each group, with chickens of northern China as a reference. A total of 233 PSGs were identified in genome-wide selection sweeps detection of the Indonesian group, among which 188 were breed-unique PSGs (Figure 2A, Supplementary Table S3). The ∼40 kb candidate region on chromosome 10 (9,640,001–9,680,000 bp), which has the highest XP-EHH score and annotates a breed-unique PSG: *CYP19A1*, was also verified by other five methods, namely, CLR, *F*st, π, Tajima’s D, and DCMS (Figures 2A, 3A). We then analyzed the pattern of haplotype homozygosity at the *CYP19A1* region in the Indonesian group in comparison with other chickens and found that haplotype sharing was much rare between Indonesian chickens and other chickens (Figure 3B). *CYP19A1* is a member of the Cytochrome P450 (CYP) superfamily and catalyzes the synthesis of estrogen via the aromatization of a ring of the androgenic steroid substrates (Nebert and Russell, 2002). In addition, three putatively candidate regions (chromosome 1: 126,240,001-126,280,000 bp, chromosome 23: 200,001-300,000 bp, chromosome 2: 28,020,001-28,060,000 bp) with high XP-EHH values (top1‰) annotate seven breed-unique PSGs. Among the seven PSGs, *WWC3* was detected using CLR, *F*st, π, Tajima’s D, and DCMS; *ARID1A*, *LIN28A*, *RPS6KA1L*, and *HMGN4* were detected using CLR, *F*st, and DCMS; *AGMO* and *MEOX2* were detected using *F*st and *π* (Supplementary Figures S1, S2). *WWC3* could suppress lung cancer invasion and metastasis (Han et al., 2017). *ARID1A* is related to lipid metabolism and immune evasion (Qu et al., 2019; Li et al., 2020). *LIN28A* is associated with dorsal root ganglion neuron apoptosis and renal fibrosis (Yu et al., 2018; Jung et al., 2020). The function of *RPS6KA1L* remains to be explored. *HMGN4* can be a promising candidate biomarker for hepatocellular carcinoma (Xia et al., 2019). *AGMO* is linked to adipogenesis and neurodevelopment (Okur et al., 2019; Fischer et al., 2021), and *MEOX2* is involved in vascular development and angiogenic response (Chen Y. et al., 2010). These candidate regions and PSGs, especially *CYP19A1*, *ARID1A*, *LIN28A*, *AGMO*, and *MEOX2*, may enable Indonesian chickens to challenge hot humid climates.

### Selection Sweeps Signals in Indian Chickens

**FIGURE 4:**
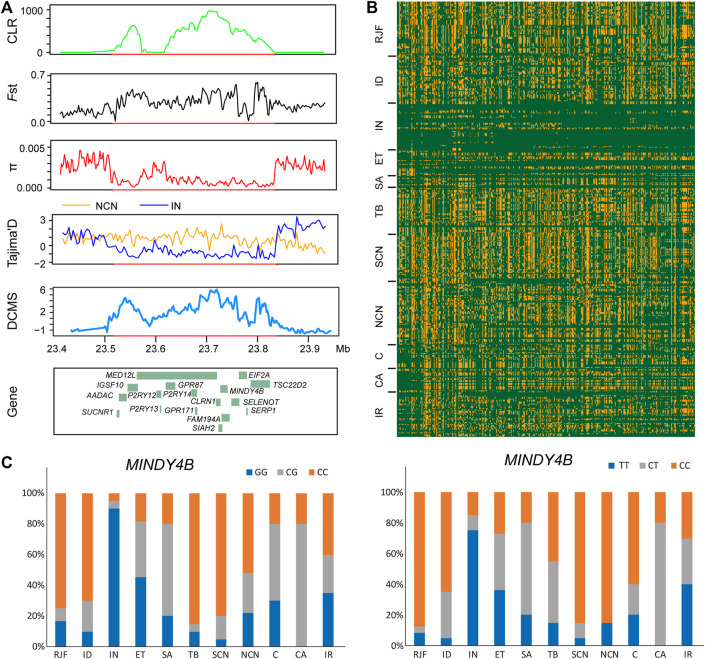
A putative selective sweep region associated with hot tolerance in Indian chickens. **(A)** five sweep statistics plotted over a 280-Kb region on chromosome 9 detected by Indian chickens. From top to bottom, the vertical axis shows the values of CLR, *F*st, π, Tajima’s D, and DCMS. **(B)** the degree of haplotype sharing around the region. **(C)** allele frequency of the mutant *MINDY4B* loci. Blue, orange, and gray represented homozygous mutant, heterozygous mutant, and homozygous wild type, respectively.

In the XP-EHH analysis of Indian chickens versus chickens of northern China, we identified 220 PSGs, among which 146 were breed-unique PSGs (Figure 2B, Supplementary Table S4). Seventeen breed-unique PSGs with the highest selective signals were identified via XP-EHH analysis to be located in a ∼280 kb candidate region on chromosome 9 (23,560,001-23,840,000 bp) (Figures 2B, 4A). In addition, this candidate region was confirmed using CLR, *F*st, π, Tajima’s D, and DCMS (Figure 4A). Compared to other groups, Indian chickens showed almost pure haplotype homozygosity in the candidate region (Figure 4B). By screening these 17 PSGs for nonsynonymous mutations representing putative functional variants, we detected two missense SNPs changed amino acids in the *MINDY4B* (p.P81A and p.H27Y). The former nonsynonymous mutation (p.P81A), in contrast to the pattern observed in other chickens, has nearly reached fixation (90%) in the Indian group. The frequency of homozygous mutant of the latter nonsynonymous mutation (p.H27Y) in Indian chickens was 75%, whereas that in other groups was no more than 40% (Figure 4C). The function of *MINDY4B* is unclear and needs to be explored. Most of the 17 PSGs are related to thermoregulation. *P2RY12* is important for platelet functions, T-cell activation, and vascular effects (vasoconstriction and vasodilation) (Gómez Morillas et al., 2021). *P2RY13* R is related to adipocyte terminal differentiation and interleukin 1β (Biver et al., 2013; Kyrargyri et al., 2020). *P2RY14* plays an important role in immunological defense systems (Katakura et al., 2020). *GPR171* plays an important role in regulating responses associated with feeding and metabolism in mice (Gomes et al., 2013). *AADAC* participates in hepatic lipid metabolism and promotes the mobilization of lipids from intracellular stores and in the liver for assembling VLDL (Trickett et al., 2001; Lo et al., 2010). *SIAH2* could promote adipogenesis (Dang et al., 2021). *SUCNR1* is implicated in the regulation of blood pressure, platelet physiology, and immune response (Gilissen et al., 2016). *SELENOT* plays an important role in brain development and function (Anouar et al., 2018). *TSC22D2* is involved in the adaptation of renal cells to hypertonicity (Fiol et al., 2007). *SERP1* stabilizes membrane proteins during stress (Yamaguchi et al., 1999). An adenoviral vector expressing short hairpin RNA targeting *GPR87* (Ad-shGPR87) had strong antitumor effects, specifically antiproliferative and proapoptotic effects (Zhang et al., 2015). Mutations in *IGSF10* could cause delayed puberty in humans (Howard et al., 2016). *MED12L* haploinsufficiency is responsible for transcriptional defect and intellectual disability (Nizon et al., 2019). *CLRN1* is essential for Cochlear Hair Cell Development (Geller et al., 2009). *FAM19A4* methylation constitutes a triage method for primary human papillomavirus (Bonde et al., 2021). *EIF2A* has a key role in the translation of hepatitis C viral Mrna (Kim et al., 2011). The candidate region and PSGs may play an important role in hot adaptation in Indian chickens.

### Selection Sweeps Signals in Saudi Arabian Chickens

**FIGURE 5:**
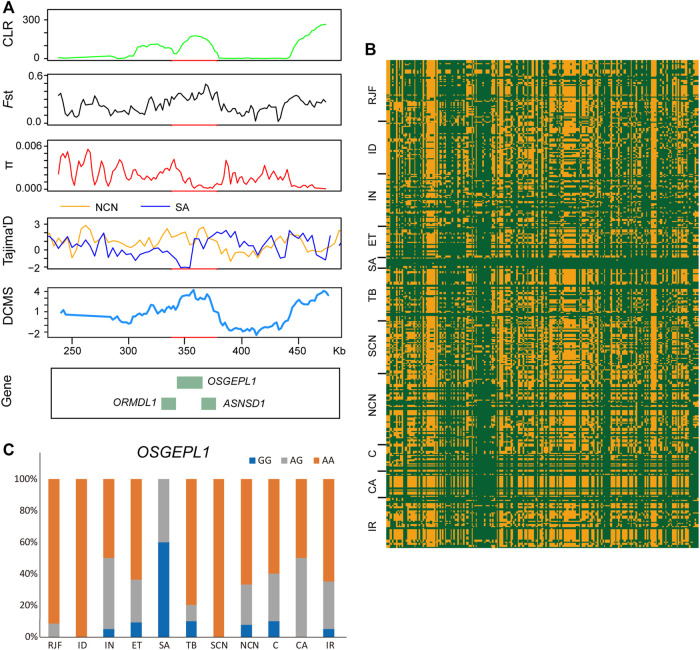
Genomic candidate regions with high selection signals in Saudi Arabian chickens. **(A)** CLR, *F*st, π, Tajima’s D, and DCMS analysis of a 40-Kb region on chromosome 7 identified by Saudi Arabian chickens. **(B)** haplotype sharing in the region. **(C)** allele frequency of the mutant *OSGEPL1* loci. The meaning of different colors is the same as that in Figure 4.

By comparing the Saudi Arabian chickens against chickens of northern China using XP-EHH analysis, we identified 272 PSGs, among which 228 were breed-unique PSGs (Figure 2C, Supplementary Table S5). We focus on four potential candidate regions that are located on chromosome 7 (340,001–380,000 bp), chromosome 1 (47,200,001-47,260,000 bp), chromosome 3 (88,180,001-88,220,000 bp), and chromosome 1 (138,160,001-138,200,000 bp). These regions contain nine breed-unique PSGs, namely, *OSGEPL1*, *ORMDL1*, *ASNSD1*, *UHRF1BP1L*, *ACTR6*, *SCYL2*, *GCLC*, *SEPT10*, and *SOWAHC*, and were also confirmed using CLR, *F*st, π, Tajima’s D, and DCMS (Figures 2C, 5A; Supplementary Figures S3, S4). In addition, the shared haplotype of these four regions was rarely observed between the Saudi Arabian group and other groups (Figure 5B). Functional annotation of variants identified a missense mutation of the *OSGEPL1* gene. The frequency of homozygous mutant of the locus in the Saudi Arabian group was 60% but less than 10% in other groups (Figure 5C). Mutation of *OSGEPL1* affects efficient t6A modification, which is essential for translational accuracy and fidelity (Zhou et al., 2020). *ORMDL1* is associated with cholesterol responses (Wang S. et al., 2015). *ASNSD1* may relate to the function of skeletal muscle (Vogel et al., 2020). *UHRF1BP1L* may be a novel candidate for myopic development (Hawthorne et al., 2013). *ACTR6* might regulate preadipocyte differentiation (Zhang T. et al., 2017). *SCYL2* plays a role in neuronal function (Pelletier, 2016). *GCLC* is indispensable for T-cell biosynthetic, and ablation of *GCLC* in T-cells impairs inflammatory responses *in vivo* (Mak et al., 2017). *SEPT10* may be a novel candidate molecule as a good indicator of paclitaxel-resistant carcinomas (Xu et al., 2012). *SOWAHC* is a potential prognostic biomarker for lung squamous cell cancer (Zhu et al., 2020). In addition, several other PSGs (*ATP1B3*, *TFDP2*, *GK5*, *XRN1*, *PEX5L*, *USP13*, *NDUFB5*, *MRPL47*, *ACTL6A*, *KIF26B*, *CDKL5*, and *HDAC4*) were identified by other four selective signal analyses and DCMS (Supplementary Figures S4, S5). *ATP1B3* is related to immune cell infiltration and immune-related cytokines expression in hepatocellular carcinoma (Lu et al., 2021). *TFDP2* affects angiogenesis, nephrogenesis, and kidney metabolic function (Köttgen et al., 2010). *GK5* exists as part of a skin-specific regulatory mechanism for cholesterol biosynthesis (Zhang D. et al., 2017). *XRN1* involves in cellular processes and development (Jones et al., 2012). *PEX5L* assists in the transport of peroxisome (Kunze et al., 2015). *USP13* has a function in regulating innate antiviral immunity (Sun et al., 2017). *NDUFB5* might act as a potential biomarker for the treatment of septic cardiomyopathy (Kang et al., 2020). A variant of *MRPL47* is a putative new risk factor for vincristine-induced peripheral neuropathy in childhood acute lymphoblastic leukemia (Abaji et al., 2018). *ACTL6A* is an oncogenic driver in head and neck squamous cell carcinoma (Saladi et al., 2017). *KIF26B* is essential for kidney development (Uchiyama et al., 2010). *CDKL5* plays a critical role in neuronal morphogenesis (Chen Q. et al., 2010). *HDAC4* relates to vascular inflammation and neuronal survival (Wang et al., 2014; Yang et al., 2018). These PSGs with different functions may allow Saudi Arabian chickens to develop physiological and genetic adaptations to the hot arid climate.

### Selection Sweeps Signals in Hot Adapted Chickens

**FIGURE 6:**
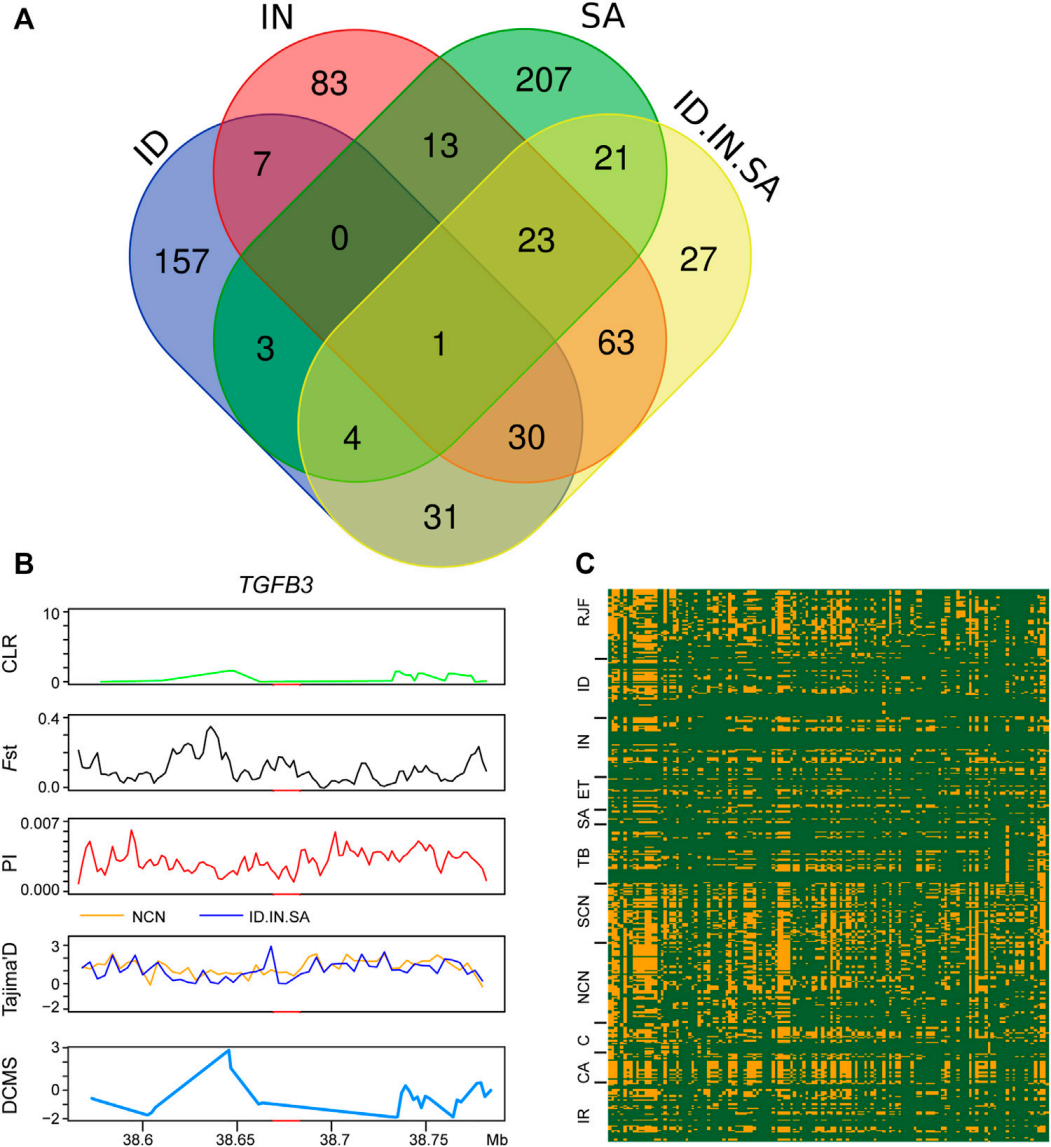
Genomic candidate gene detected XP-EHH analyses of four chicken groups. **(A)** venn diagram showing the PSGs overlap among XP-EHH_ID-to-NCN_, XP-EHH_IN-to-NCN_, XP-EHH_SA-to-NCN_, and XP-EHH_ID.IN.SA-to-NCN_. **(B)** CLR, Fst, π, Tajima’s D, and DCMS analysis of TGFB3 identified by XP-EHH analyses of four chicken groups.

We also conducted an XP-EHH analysis with all three groups (Indonesian, Indian, and Saudi Arabian chickens) as a single candidate group against chickens of northern China and identified 200 PSGs. Among the 200 PSGs, 66 PSGs, 117 PSGs, and 49 PSGs were found in individual XP-EHH analyses of the Indonesian, Indian, and Saudi Arabian groups, respectively (Figure 2D, Figure 6A and Supplementary Table S6). Only one gene, the *TGFB3* gene, was detected using both this combined XP-EHH analysis and three individual analyses of Indonesian, Indian, and Saudi Arabian chickens. Futhermore, this PSG was not verified using other selection sweeps analyses, haplotype analysis, and missense mutation (Figures 6B,C). In addition, based on the XP-EHH score of three groups combination versus chickens of northern China, the top 36 potential candidate regions, including 92 PSGs, were chosen to perform other selection sweep analyses, CLR, Fst, π, Tajima’s and DCMS. Just two potential candidate regions (chromosome 5: 41,000,001–41,040,000 bp and chromosome 4: 54,640,001–54,720,000 bp) were confirmed using CLR, *F*st, π, Tajima’s D, and DCMS (Supplementary Figure S6). These candidate regions contain four PSGs, namely, *GTF2A1*, *TSHR*, *PDE5A*, and *FABP2* (Supplementary Figure S6). *GTF2A1* is related to the function of the ovary and uterus, hence influencing egg production (Yuan et al., 2015). *TSHR* has biological significance in metabolic regulation and reproduction process (Nakao et al., 2008). *PDE5A* is important in the regulation of vascular tone (Gebska et al., 2011). *FABP2* may act as a lipid-sensing component of energy homeostasis (Vassileva et al., 2000). The above regions and PSGs may be important candidates in three different tropical local populations and should be important targets for further in-depth research.

## Discussion

Chickens, which are among the most important domestic animals, are widely distributed around the world and raised in the largest numbers (Lawler, 2016). Chickens can provide high-quality and cheap animal protein. However, with the changes in climatic conditions, production environment, and customer demand, poultry breeding goals are shifting from merely improving production traits to also incorporating the traits of environmental resilience, disease resistance, and animal welfare (Muchadeyi and Dzomba, 2017). Indigenous chicken breeds with strong adaptability and stress resistance represent excellent genetic resources, which can be used for breeding improvement to meet the emerging demand. However, there are limited systematic studies on the hot adaptation of indigenous chickens that live in different tropical climate regions. In this study, we analyzed the population structure and selection signature of indigenous chickens from different tropical climate regions (Indonesia, India, and Saudi Arabia) to elucidate the genetic mechanisms of hot adaptation in indigenous chickens.

In population structure analysis, we found that chickens of northern China had a remote relationship with three other types of chickens, whereas Saudi Arabian chickens harbor a closer relationship with Indian chickens (Figure 1). XP-EHH-based selection sweep analysis has detected strong signals related to hot adaptation in Indonesian, Indian, and Saudi Arabian chickens. We also performed the XP-EHH analysis of these three groups as a single group. Although TGFB3 was detected using all four XP-EHH analyses (three individual analyses of Indonesian, Indian, and Saudi Arabian chickens and one combined analysis), it was not verified by other selection sweeps analyses and haplotype analysis (Figure 6). Only two regions identified using XP-EHH analyses (three groups combination versus chickens of northern China) were also verified using CLR, *F*st, *π*, and Tajima’s D and DCMS (Supplementary Figure S6). It seems that the more reliable PSGs that can be verified using other methods are all breed-unique PSGs and that each type of indigenous chicken inhabiting different tropical climate zones has developed its own unique genetic mechanisms for hot adaptation. Such adaptation to local environments has been reported in wheat landraces in Iran and Pakistan (Hanif et al., 2021). Hence, each indigenous breed can be used for local breed improvement.

Focusing on the selected signals unique to each group, we found that several PSGs are related to the nervous system, vasoconstriction and vasodilation, immune system, lipid metabolism, and kidney function. The nervous system plays an important role in thermoregulatory responses (Boulant and Dean, 1986). *LIN28A* and *AGMO* in Indonesian chickens; *SELENOT* in Indian chickens; and *SCYL2*, *CDKL5*, and *HDAC4* in Saudi Arabian chickens are all related to the regulation of the nervous system. Vasoconstriction and vasodilation control blood flow and consequently affect heat loss and stress (Collier and Collier, 2012). *MEOX2* in Indonesian chickens; *P2Y12* in Indian chickens; *TFDP2* and *HDAC4* in Saudi Arabian chickens are all linked to vascular function. Climate changes increase the spread of pathogens and parasites, which challenge the immune system of humans and animals (Uddin et al., 2010; Indhumathi and Kumar, 2021). *ARID1A* in Indonesian chickens; *P2Y12*, *P2RY13*, *P2RY14*, and *SUCNR1* in Indian chickens; *GCLC*, *ATP1B3*, and *USP13* in Saudi Arabian chickens are associated with immune functions. Heat stress affects feed intake and adipogenesis. *CYP19A1*, *ARID1A*, and *AGMO* in Indonesian chickens; *P2RY13*, *AADAC*, and *SIAH2* in Indian chickens; *ORMDL1* and *ACTR6* in Saudi Arabian chickens are connected with lipid metabolism. Kidney development and function are essential for heat acclimation (Chaffee et al., 1969). *LIN28A* in Indonesian chickens; *TSC22D2* in Indian chickens; *TFDP2* and *KIF26B* in Saudi Arabian chickens are involved in kidney function. Altogether, the chickens from different tropical climate zones seem to be evolved in parallel by taking various pathways involving different complements of genes to cope with specific tropical climates. Such a phenomenon has been observed in Tibetan chickens. Different lineages of Tibetan chickens harbor distinct gene suites in their high-altitude adaptation process, and few potential candidate genes were found to overlap for the cold-tolerant chickens from different regions (Wang M.-S. et al., 2015; Xu et al., 2021).

The sample size in our study was limited, in particular, there are only five Saudi Arabian chickens. Thus, we employed four classic methods (CLR, *F*st, *π*, and Tajima’s D) and the DCMS method to identify whole-genome selection sweep signals for three chicken breeds from different tropical climates and hoped to capture the most relevant selective regions and genes. In addition, we conducted haplotype analysis among candidate groups, reference groups, and other seven groups to further verify candidate regions and PSGs. In the future, more samples from diverse breeds will be collected, sequenced, and analyzed, and RNA-seq and further functional experiments will be performed to further understand the genetic mechanisms of adaptations to tropical climates in chickens.

In sum, our selective sweep analyses have revealed a variety of important candidate regions and PSGs associated with genetic adaptations of chickens to tropical rainforest climate, tropical monsoon climate, and tropical desert climate. Such findings have laid a foundation for breeding improvement in different tropical climates.

## Data Availability

The whole genome re-sequencing data of this study are available in the NCBI Sequence Read Archive (BioProject PRJNA232548, PRJNA306389, PRJNA453469, PRJNA720223, PRJNA202483, PRJNA241474) and the ChickenSD database (http://bigd.big.ac.cn/chickensd/).
