# Supplementary material for: Genomic Analyses for Selective Signatures and Genes Involved in Hot Adaptation Among Indigenous Chickens From Different Tropical Climate Regions
Source: Front Genet. 2022 Jul 22;13:906447. doi: 10.3389/fgene.2022.906447 (PMC9377314; doi:10.3389/fgene.2022.906447)
Supplement: Supplementary file 1 [file DataSheet1.docx]

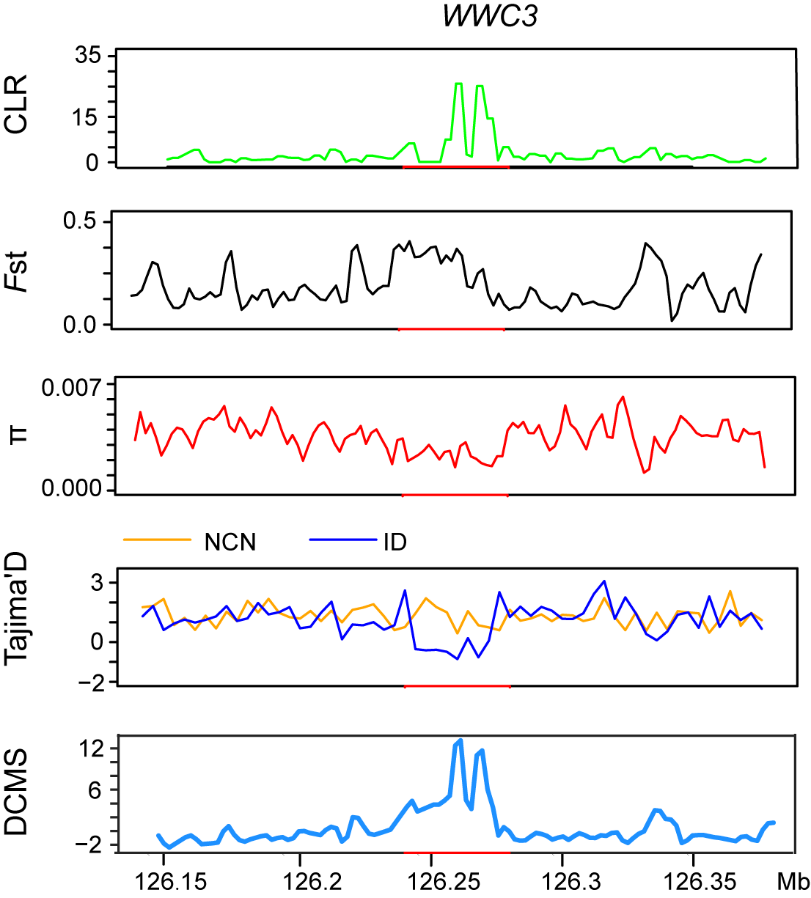


**Supplementary Figure S1.** Selective signature on *WWC3* in ID chickens.


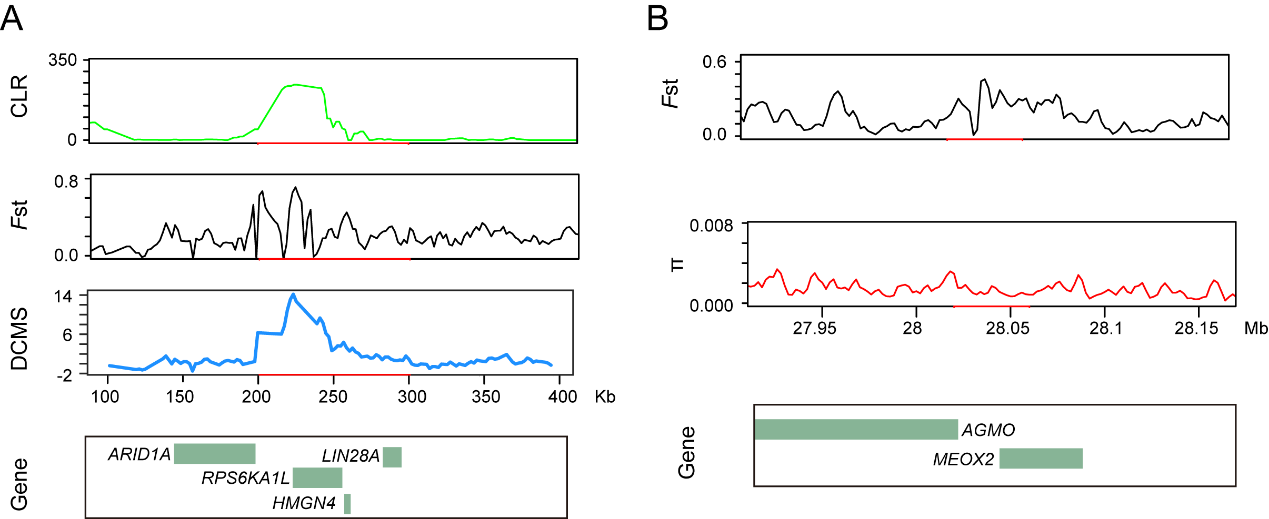


**Supplementary Figure S2.** Two candidate selective sweep regions in ID chickens.


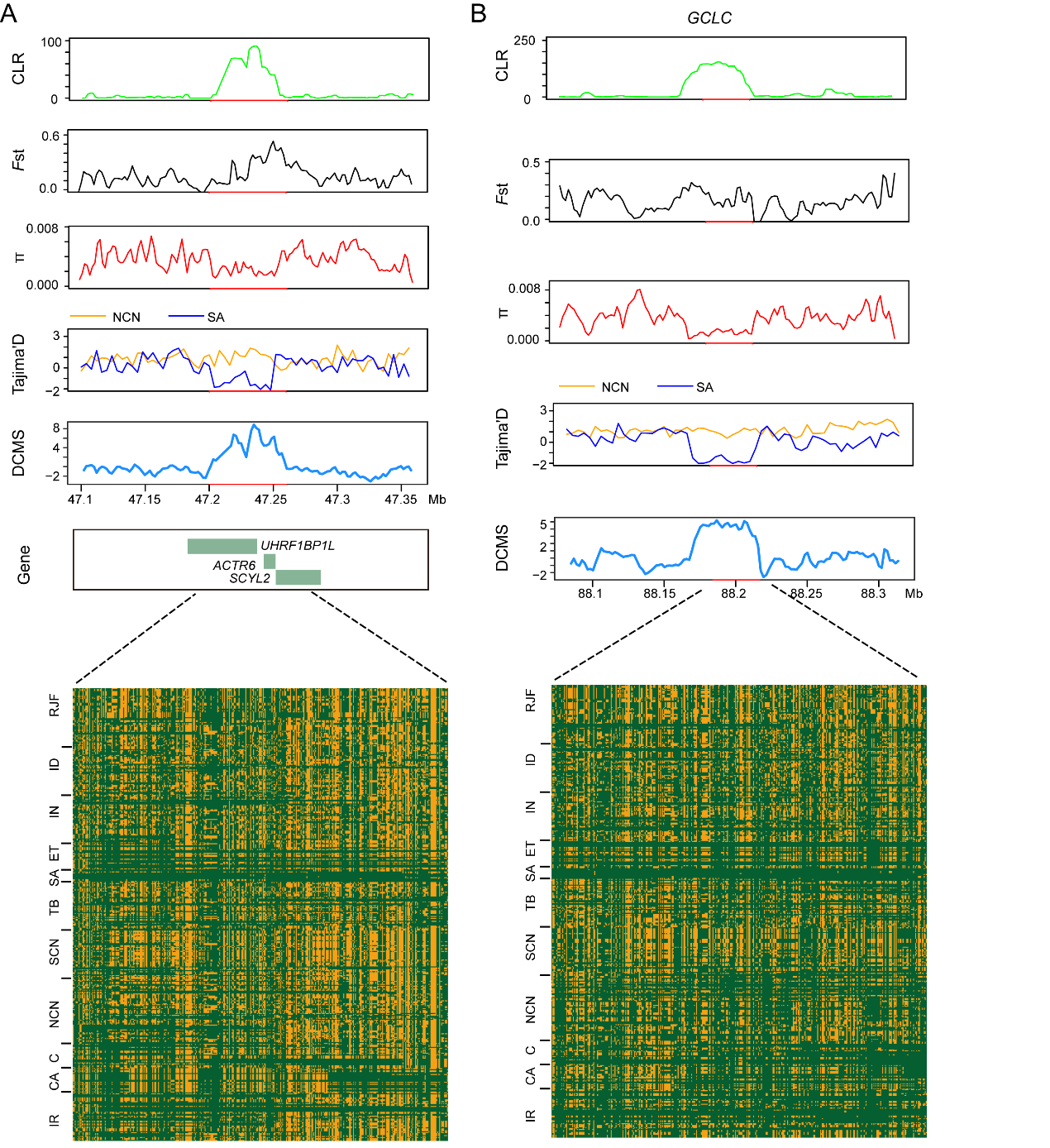


**Supplementary Figure S3.** Selective signature and haplotype analysis on two candidate regions of SA chickens.


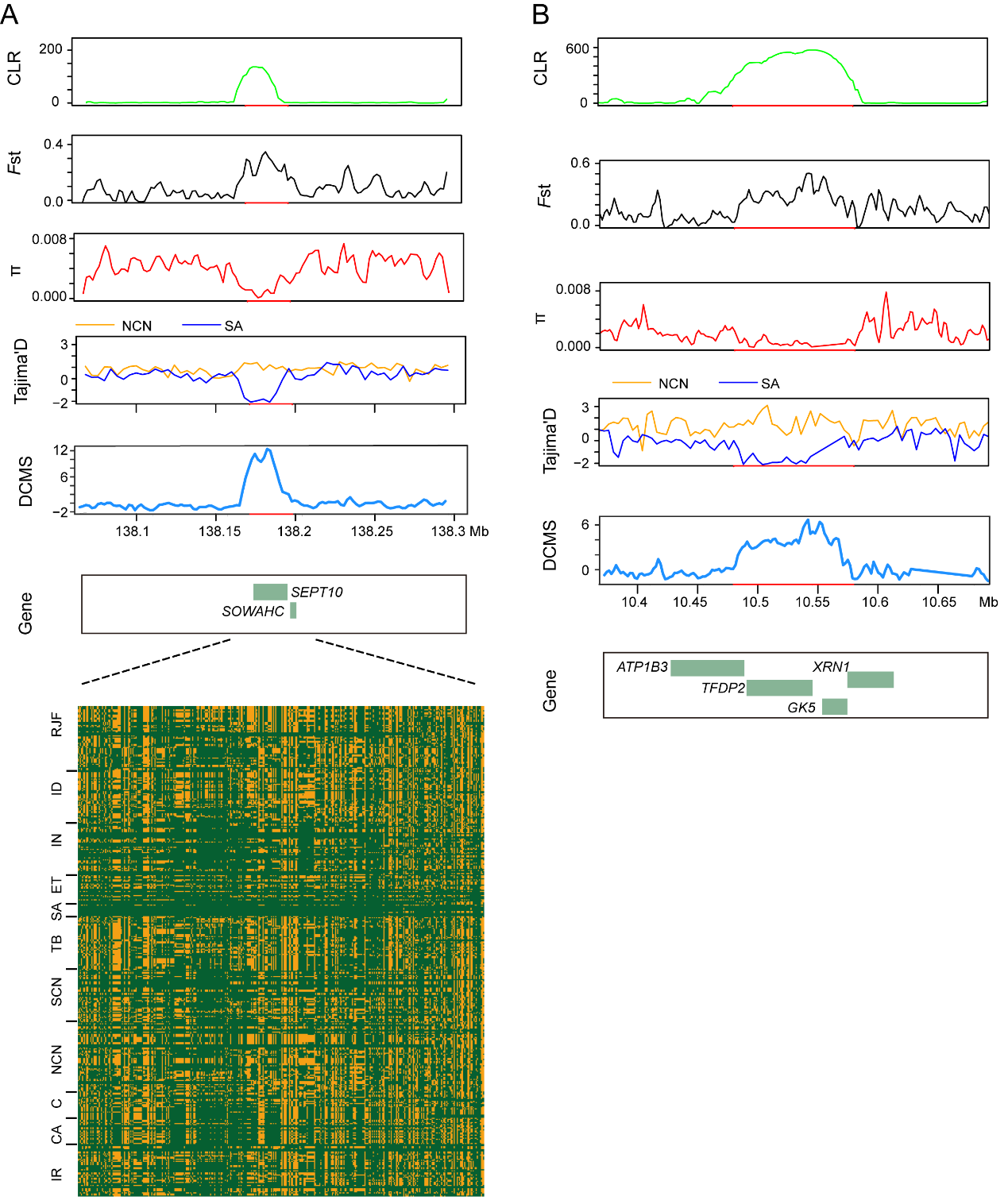


**Supplementary Figure S4.** Examples of two candidate regions on SA chickens.


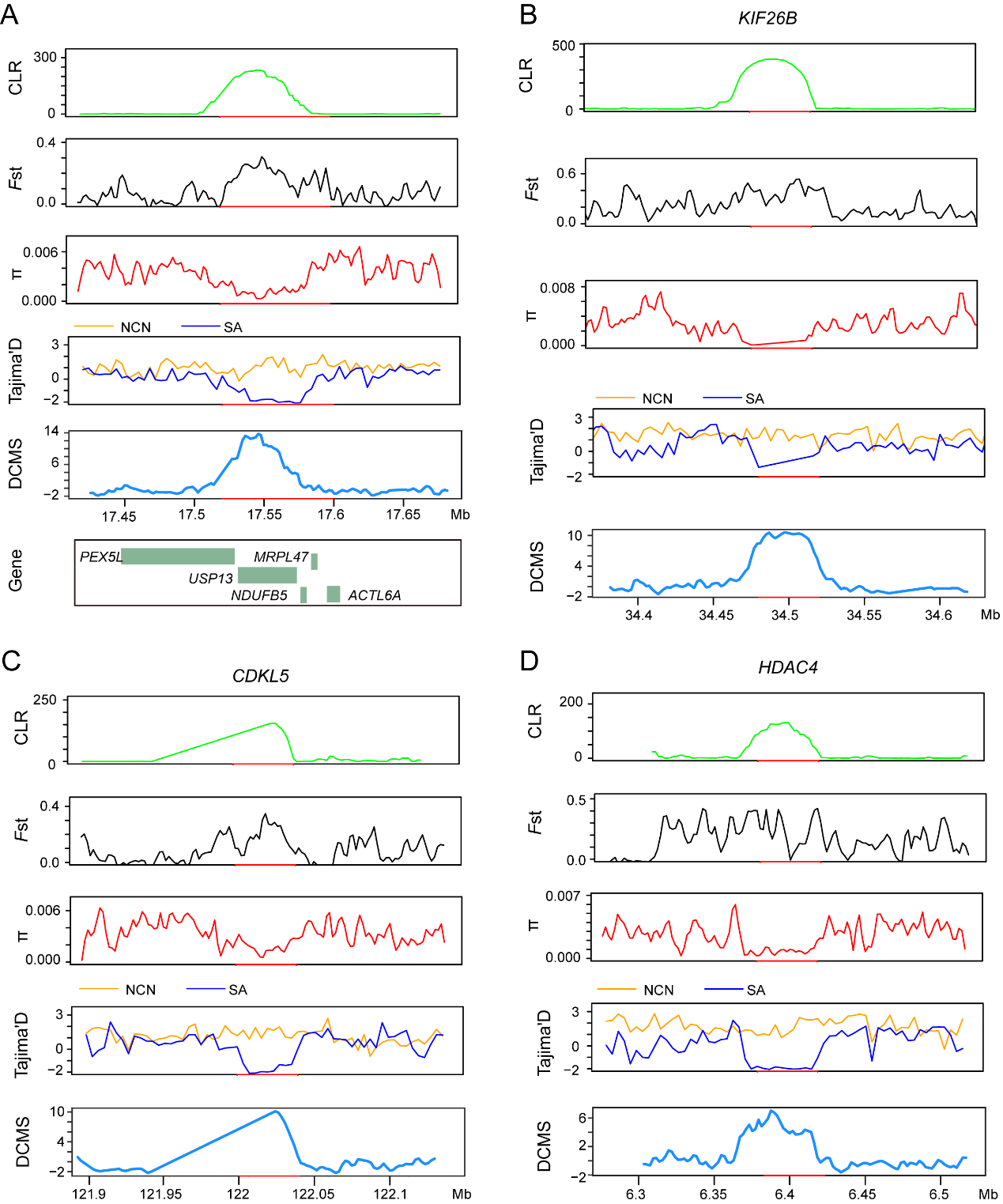


**Supplementary Figure S5.** Selective signature of candidate selective regions on SA chickens.


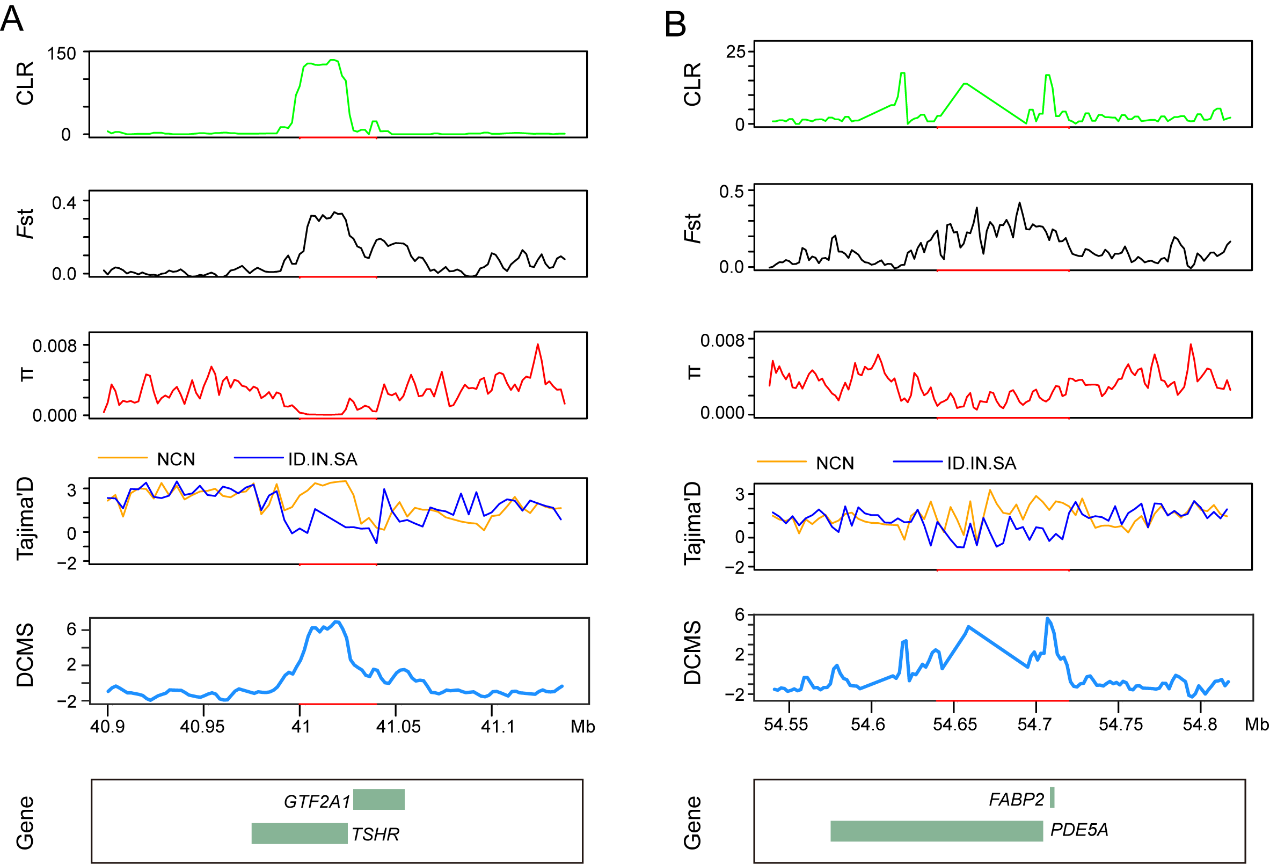


**Supplementary Figure S6.** Selective signature on two candidate regions of ID.IN.SA chickens.
